# Phytonutrient-Enriched Prebiotic Mixture Primes the Gut Environment to Enhance Probiotic Efficacy: Ex Vivo Screening and a Human Clinical Trial

**DOI:** 10.3390/biology15131006

**Published:** 2026-06-25

**Authors:** Hyo-Jin Lee, Dong Ho Suh, Sunyoung Lee, Wilhelm H. Holzapfel, Yosep Ji, Matthew K. Runyon, Hae Jo, Jung-Yoon Hur, Ri Ryu, Eun Sung Jung

**Affiliations:** 1 HEM Pharma Inc., Suwon 16229, Republic of Korea; hjlee@hempharma.bio (H.-J.L.); dhsuh@hempharma.bio (D.H.S.);; 2 Department of Advanced Convergence, Handong Global University, Pohang 37554, Republic of Korea; 3 Amway Research and Development, Ada, MI 49355, USA

**Keywords:** phytonutrient-enriched prebiotic mixture, short-chain fatty acids, butyrate production, ex vivo, PMAS

## Abstract

Phytonutrient-enriched prebiotic mixtures (PEPs), composed of phytonutrients and prebiotics serving as substrates for gut microbes, are recognized for their potential to modulate gut microbial metabolic activity. However, direct evidence of enhanced effects following co-administration with probiotics remains limited. Using a three-phase design integrating ex vivo evaluation and clinical validation, we assessed how PEP components influence microbial responses and whether co-administration with probiotics enhances these effects. PEP components increased acetate, butyrate, total short-chain fatty acids (SCFAs), and lactate, with fiber-rich components showing the strongest effects (all q < 0.0001 relative to negative control). Co-treatment with probiotics further enhanced butyrate and total SCFAs in a dose-dependent manner. In a randomized clinical study, all groups showed increases in fecal metabolites, with the combined group exhibiting the greatest increases in butyrate (+6.0 µmol/g, ~1.5-fold, *p* < 0.05) and total SCFAs (+22.9 µmol/g, ~1.3-fold, *p* < 0.05). Participants with constipation-type stool patterns shifted toward normal stool types across all groups. These findings support the utility of combined PEP and probiotic interventions for enhancing microbiome-derived metabolic activity.

## Simple Summary

This study investigated whether a phytonutrient-enriched prebiotic mixture (PEP), designed to support beneficial gut microbes, can improve gut microbial activity and whether combining it with probiotics provides additional benefits. Many dietary components are not fully absorbed and instead reach the gut, where they are metabolized by microbes into compounds that influence health. However, their combined effects with probiotics remain unclear. To address this, we first evaluated different PEP components using human stool samples in an ex vivo system, and then confirmed the findings in a human clinical study. PEP increased the levels of beneficial metabolites, particularly butyrate and total short-chain fatty acids (SCFAs). Greater increases were observed when PEP was co-administered with probiotics, especially at higher probiotic doses. In the clinical study, all groups showed improvements, but the combined PEP and probiotic group exhibited the greatest increases in beneficial metabolites. Participants with constipation-type stool patterns also shifted toward normal stool types. These findings suggest that combining specific dietary components with probiotics may enhance microbiome-derived metabolic activity and provide a basis for future synbiotic strategy development.

## 1. Introduction

Interest in microbiome-targeted nutritional strategies has increased substantially, particularly as dietary components are now understood to exert their effects not only through direct host absorption but also through microbiome-mediated metabolic transformation [1]. Among these, phytonutrients, especially plant-derived polyphenols, have received considerable attention as bioactive compounds capable of modulating the intestinal environment through interactions with the gut microbiota [2]. A substantial proportion of dietary polyphenols escapes absorption in the small intestine and reaches the colon, where they are extensively metabolized by gut microbes into bioactive, low-molecular-weight compounds [3]. This microbiome-dependent biotransformation is increasingly recognized as a key determinant of the functional impact of phytonutrients on host physiology, including antioxidant activity and maintenance of intestinal barrier integrity [4].

Beyond phytonutrients, microbiome-directed interventions have traditionally focused on prebiotics and probiotics as complementary strategies to modulate gut microbial activity. Prebiotics, defined as substrates selectively utilized by host microorganisms that confer a health benefit, provide fermentable carbon sources that drive microbial metabolism toward beneficial outputs such as short-chain fatty acids (SCFAs) [5]. Probiotics, in contrast, introduce live microorganisms with functional potential to influence microbial composition and metabolic activity [6]. The combined administration of these components, often referred to as synbiotics, is hypothesized to enhance microbiome function by aligning substrate availability with microbial metabolic capacity [7]. However, despite increasing interest, most studies have evaluated individual components or limited combinations, and evidence remains insufficient to determine whether multi-component strategies consistently yield additive or synergistic metabolic benefits under physiologically relevant conditions [8].

A central functional output of microbiome-directed interventions is the production of fermentation-derived metabolites, particularly SCFAs, which serve as key mediators linking diet, microbial activity, and host physiology [9]. SCFAs, including acetate, propionate, and butyrate, are generated through anaerobic fermentation of non-digestible carbohydrates and exert diverse physiological effects. Acetate contributes to systemic energy metabolism and serves as a substrate for peripheral tissues [10], while propionate is associated with gut–liver metabolic pathways and has been studied in the context of metabolic regulation [11]. Butyrate, however, is of particular importance as the primary energy source for colonocytes and a critical regulator of intestinal barrier function and immune homeostasis [12]. Importantly, SCFA production occurs within a complex microbial metabolic network involving cross-feeding interactions. In this context, lactate, often considered a transient fermentation intermediate, plays a critical role as a precursor for butyrate production through lactate-utilizing pathways. Acetate can act as a co-substrate in this conversion, supporting the transformation of lactate into butyrate and thereby influencing the overall metabolic balance within the microbial community [13]. These interconnected pathways highlight the importance of evaluating not only SCFAs but also upstream metabolites such as lactate when assessing microbiome-mediated metabolic responses.

Despite growing recognition of these metabolic mechanisms, several limitations remain in current research. Many studies rely on single-ingredient interventions or simplified combinations, which do not fully capture the complexity of real-world dietary formulations or microbiome interactions [8]. In addition, substantial inter-individual variability in microbiome composition leads to heterogeneous metabolic responses, complicating the interpretation of intervention outcomes [14]. Furthermore, conventional experimental approaches face practical challenges: direct clinical trials are resource-intensive and limited in scalability, while existing in vitro fermentation models often lack the throughput or personalization needed to systematically evaluate multiple components across diverse microbiomes [15]. To overcome these limitations, advanced ex vivo platforms capable of simulating individualized gut environments while enabling high-throughput screening have emerged as valuable tools. The Personalized Pharmaceutical Meta-Analytical Screening (PMAS) system has been developed to reproduce key features of the intestinal environment and capture inter-individual variability in microbiome responses, thereby providing a translational bridge between controlled experiments and clinical validation [16].

In this study, we evaluated a phytonutrient-enriched prebiotic mixture (PEP), composed of phytonutrients and prebiotics that serve as substrates for beneficial gut microbes, and its combination with probiotics to investigate potential synbiotic effects on microbiome-derived metabolic activity. The PEP was rationally designed to combine, within one formulation, four complementary classes of microbiome-active ingredients (Tables S1 and S2): (i) fermentable prebiotic fibers (fructooligosaccharides and apple fiber) as saccharolytic substrates for SCFA, particularly butyrate, production [5]; (ii) polyphenol- and phytochemical-rich botanicals (e.g., ginger, turmeric, cinnamon, leafy greens, and fruit and vegetable blends) that the microbiota biotransform into bioactive metabolites, reshaping community structure and SCFA output [2,4]; (iii) micronutrients (vitamins B1, B2, and C, and zinc); and (iv) heat-treated probiotic components (*Lactiplantibacillus plantarum* and *Bifidobacterium longum* subsp. *longum*). Because fermentable fiber alone does not capture the community-level effects of real-world formulations, we paired it with polyphenol-rich phytonutrients to prime the gut toward butyrogenic cross-feeding and cooperative action with co-administered probiotics. Using SCFAs and lactate as primary functional readouts, we applied a three-phase framework integrating ex vivo evaluation with clinical validation. This approach enabled us to examine component-dependent metabolic responses to PEP, characterize interactions between PEP and probiotics, and assess whether these effects translate to humans in a randomized clinical study. Ultimately, we aimed to determine whether co-administration enhances the production of metabolites, while establishing a scalable framework for microbiome-targeted nutritional strategies.

## 2. Materials and Methods

### 2.1. Study Overview and Experimental Framework

**Figure 1:**
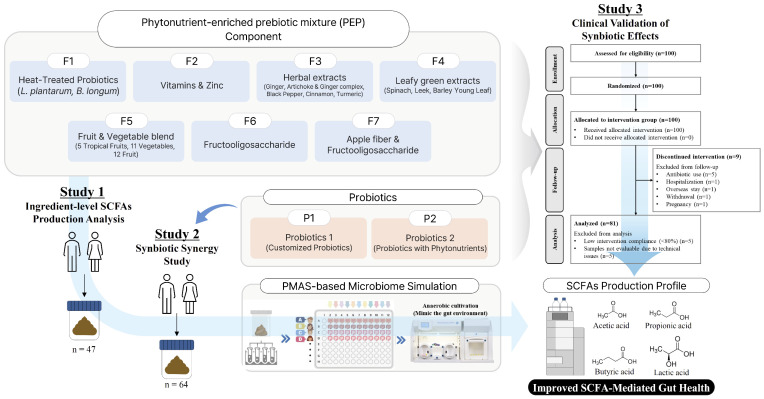
Schematic overview of the three-study design and experimental workflow. The study consisted of three sequential phases. First, an ex vivo evaluation using the PMAS-based microbiome simulation system assessed multiple components (F1–F7) of a phytonutrient-enriched prebiotic mixture (PEP) for their effects on short-chain fatty acids (SCFAs) production. Second, an ex vivo study evaluated the combined effects of PEP and probiotics, including customized probiotics (P1) and phytonutrient-associated probiotics (P2). Third, a 4-week randomized clinical study was conducted to validate these findings in humans, in which participants received PEP alone, probiotics alone, or their combination. Fecal samples were collected before and after the intervention, and SCFAs production profiles were analyzed.

This study was designed to evaluate the functional impact of a phytonutrient-enriched prebiotic mixture (PEP), probiotics, and their combined administration on gut microbiome activity, with a primary focus on microbiome-derived metabolite production. The overall experimental framework followed a translational design integrating ex vivo microbiome simulation and human clinical validation, as illustrated in Figure 1. The study was conducted in three sequential phases: (i) an initial ex vivo evaluation of microbiome responses to PEP components (Study 1), (ii) an ex vivo evaluation of interactions between PEP and probiotics (Study 2), and (iii) a randomized clinical trial to validate these findings (Study 3). Across all phases, metabolite production profiles were used as key functional readouts reflecting microbiome-mediated metabolic activity and their potential contribution to gut health. The baseline characteristics of the fecal donors were summarized in Table 1.

### 2.2. The Design of Study 1: Ex Vivo Evaluation of Microbiome Responses to PEP Components

Study 1 was conducted to characterize variability in microbiome-mediated metabolic responses to different PEP components using an ex vivo evaluation approach. A total of 47 fecal samples obtained from healthy donors were used to capture inter-individual differences in microbial fermentation capacity. The PEP tested in this study consisted of multiple functional components, including heat-treated probiotic strains (*Lactobacillus plantarum* and *Bifidobacterium longum*), micronutrients such as vitamins and zinc, a diverse set of herbal and leafy green extracts, fruit- and vegetable-derived phytonutrient blends, and fermentable substrates including fructooligosaccharides (FOS) and apple fiber. These components were combined into several PEP compositions (F1–F7), as detailed in Table S1, to assess how variation in component composition influences microbiome-derived metabolite production. Each fecal sample was treated with the different PEP compositions under controlled anaerobic conditions, and metabolite production profiles were quantified following incubation. Rather than identifying a single optimal composition, this approach enabled the characterization of response patterns across individuals, providing insight into how microbiome composition influences fermentation dynamics and metabolite production.

### 2.3. The Design of Study 2: Ex Vivo Evaluation of PEP–Probiotic Interactions

Study 2 was designed to investigate whether PEP exerts enhanced effects when combined with probiotic treatments. Fecal samples from 64 independent healthy donors were used to evaluate microbiome responses under different treatment conditions. In addition to PEP alone, this study included multiple probiotic formulations, comprising both customized probiotic blends and commercially available products containing phytonutrients, as summarized in Table S2. Here, P1 denotes a personalized-probiotic treatment arm comprising six individually tailored lactic acid bacteria (LAB)-based formulations (Probiotic mixtures 1–6, Table S2), which are analyzed collectively under the single label “P1” because they share this common personalized-probiotic concept; P2 denotes a single phytonutrient-containing LAB product. All of these formulations were used in both Study 2 (ex vivo) and Study 3 (clinical). The combined treatments were designed to capture a wide range of microbiome responses and product-specific effects. All treatments were applied at concentrations scaled to reflect physiologically relevant intake levels and adjusted to the conditions of the ex vivo system. By comparing metabolite production profiles across PEP-only, probiotic-only, and combination treatments, this phase evaluated whether combined treatment enhances microbiome-derived metabolic activity compared to individual treatments.

### 2.4. Ex Vivo Microbiome Simulation and Sample Preparation

All ex vivo experiments in Study 1 and Study 2 were conducted using the Personalized Pharmaceutical Meta-Analytical Screening (PMAS) system, an ex vivo platform designed to simulate the human gut environment under controlled laboratory conditions. The PMAS system enables the evaluation of microbiome-derived metabolic responses using individual fecal samples while preserving inter-individual variability. This platform is based on patented technologies (U.S. Patent No. 11237172 [17]; KR Patent No. 10-2124474 and 10-2227382 [18]) that reproduce key physiological features of the intestinal environment, including strict anaerobic conditions, gut-mimicking culture media, and controlled mixing dynamics that simulate intestinal peristalsis. The microplate-based format allows parallel assessment of multiple treatments and efficient profiling of microbiome-derived metabolites. Each fecal sample was quantitatively homogenized in PMAS culture medium containing L-cysteine hydrochloride, mucin, hemin, resazurin sodium salt, sodium chloride, sodium hydrogen phosphate, and potassium chloride (all reagents purchased from Sigma-Aldrich, St. Louis, MO, USA). The homogenized samples were filtered using sterile filter bags (Whirl-Pak^®^, Nasco Sampling, Fort Atkinson, WI, USA) to obtain clarified fecal supernatants. For control conditions, untreated supernatants were aliquoted into flat-bottom 96-well plates (SPL Life Sciences, Pocheon, Republic of Korea). For treatment conditions, filtered fecal supernatants were exposed to PEP, probiotic products, or their combinations according to the experimental design. Product concentrations in the PMAS system were determined using a daily intake-equivalent approach. Briefly, the recommended daily intake of each product was converted according to the relative amount of fecal material used in the ex vivo culture system, based on the average daily fecal output of a healthy adult. This approach was designed to approximate physiologically relevant exposure conditions while enabling comparative evaluation of microbiome-derived metabolic responses across treatments. All the products for testing used in this study were provided by Amway Korea (Seoul, Republic of Korea), a regional subsidiary of Amway Corporation (Ada, MI, USA). Each condition was tested in biological duplicates, with samples collected at baseline (0 h, unincubated) and after incubation for 24 h at 37 °C under anaerobic conditions. Incubation was performed in an oxygen-free environment continuously supplied with a gas mixture of N_2_, CO_2_, and H_2_ using an anaerobic workstation (Whitley A95 Workstation, Don Whitley Scientific, Bradford, UK). Following incubation, all samples were immediately stored at −80 °C to quench microbial activity and preserve metabolite profiles for subsequent analysis.

### 2.5. The Design of Study 3: Clinical Validation of Synbiotic Effects

To validate the ex vivo findings, a randomized, open-label, parallel-group clinical trial was conducted in healthy adult participants. The study protocol was approved by the Public Institutional Review Board designated by the Ministry of Health and Welfare (IRB No. P01-202512-01-048, 29 December 2025), and all procedures were performed in accordance with the Declaration of Helsinki. Written informed consent was obtained from all participants prior to enrollment.

A total of 100 healthy adults were recruited and randomly assigned to one of three primary groups for a 4-week period. Participants in the PEP group received 5 g/day of PEP, while those in the probiotic group received 1.5 g/day of probiotic products. The combination group received both treatments concurrently. To account for variability in probiotic efficacy and to evaluate broader applicability, participants assigned to probiotic-containing groups were initially distributed across different probiotic types, including customized probiotics and probiotics containing phytonutrients, as detailed in Table S2. However, as the primary objective of this study was to evaluate the overall effect of probiotics rather than product-specific differences, all probiotic groups were combined for data analysis. During the study period, nine participants discontinued due to antibiotic use (*n* = 5), hospitalization (*n* = 1), overseas travel (*n* = 1), withdrawal of consent (*n* = 1), or pregnancy (*n* = 1). An additional ten participants were excluded from the final analysis due to low compliance (<80%, *n* = 5) or non-evaluable samples resulting from technical issues (*n* = 5). Consequently, data from 81 participants were included in the final analysis. Fecal samples and gastrointestinal health-related questionnaires were collected at baseline and after the intervention. Eligibility criteria excluded individuals with gastrointestinal disorders, recent antibiotic use, or regular intake of prebiotic or probiotic supplements to minimize confounding effects.

### 2.6. Quantification of Short-Chain Fatty Acids and Lactate

Short-chain fatty acids and lactate were quantified as a primary functional readout of microbial metabolic activity. For ex vivo samples, culture supernatants were collected following anaerobic incubation, centrifuged at 3153× *g*, and filtered using a 0.22 µm filter plate (Multiscreen^®^ 96-well plate with hydrophilic PVDF membrane, Merck, Darmstadt, Germany) prior to analysis. For clinical samples, 0.15 g of fecal material collected at baseline and post-intervention was transferred into 2 mL tubes and mixed with 500 µL of HPLC-grade water (Samchun Pure Chemical Co., Ltd., Pyeongtaek, Republic of Korea). After thorough vortexing, samples were centrifuged at 3153× *g* for 10 min at room temperature. The resulting supernatants were filtered using a 0.22 µm filter plate into a new collection plate. Subsequently, 10 µL of 1 M methanol (Sigma-Aldrich, St. Louis, MO, USA) was added as a reference compound to monitor injection reproducibility and retention time stability. Methanol produced a distinct and well-resolved peak under the chromatographic conditions and did not interfere with the detection of target metabolites.

Quantitative analysis was performed using a liquid chromatography system equipped with a refractive index detector (LC-RID; Shimadzu Corporation, Kyoto, Japan). Chromatographic separation was achieved using a Rezex ROA-Organic Acid H^+^ column (Phenomenex, Torrance, CA, USA) with 0.01 M sulfuric acid (Daejung Chemicals & Metals Co., Ltd., Siheung, Republic of Korea) in water (Samchun Pure Chemical Co., Ltd., Pyeongtaek, Republic of Korea) as the mobile phase. The analysis was conducted under isocratic conditions at a flow rate of 1.0 mL/min for 8 min, with the column oven temperature maintained at 75 °C. The analysis included the quantification of major fermentation-derived metabolites, namely acetic acid, propionic acid, butyric acid, and lactate, thereby capturing both canonical short-chain fatty acids and upstream fermentation intermediates. Standard solutions (Sigma-Aldrich, St. Louis, MO, USA) were prepared by serial dilution using the mobile phase, and metabolite concentrations in the samples were determined based on corresponding calibration curves.

### 2.7. Statistical Analysis

Statistical analyses were performed using R software (version 4.3.2). For ex vivo experiments, differences between treatment conditions and negative control (NC) were assessed using the paired Wilcoxon signed-rank test. To account for multiple comparisons, *p*-values were adjusted using the Benjamini–Hochberg (BH) procedure, and statistical significance was defined based on the adjusted q-values. For clinical data, comparisons between baseline and post-intervention measurements were performed using a paired *t*-test. Statistical significance was defined based on two-tailed *p*-values.

For data visualization, statistical significance was denoted using asterisks as follows: for ex vivo analyses, significance versus NC based on BH-adjusted paired Wilcoxon signed-rank tests was indicated as q < 0.05 (*), q < 0.01 (**), q < 0.001 (***), and q < 0.0001 (****). For clinical pre–post comparisons, significance based on paired *t*-tests was indicated as not significant (ns), *p* < 0.05 (*), *p* < 0.01 (**), *p* < 0.001 (***), and *p* < 0.0001 (****).

The butyrogenic conversion index (BCI) was calculated as follows: BCI = (acetate + lactate)/(acetate + propionate + butyrate + lactate). In this study, BCI was used as an exploratory index representing the relative contribution of acetate and lactate to the total measured organic acid pool. Because acetate and lactate have been reported as important substrates and intermediates in microbial cross-feeding networks associated with butyrate production [13], the index was used to summarize acetate/lactate-associated metabolic balance within the ex vivo system. Therefore, BCI should be interpreted as an inference-based summary index rather than a direct measure of biochemical conversion, pathway activity, or metabolic flux.

## 3. Results

### 3.1. Ex Vivo Evaluation of Microbiome-Derived Metabolic Responses to PEP Components

**Figure 2:**
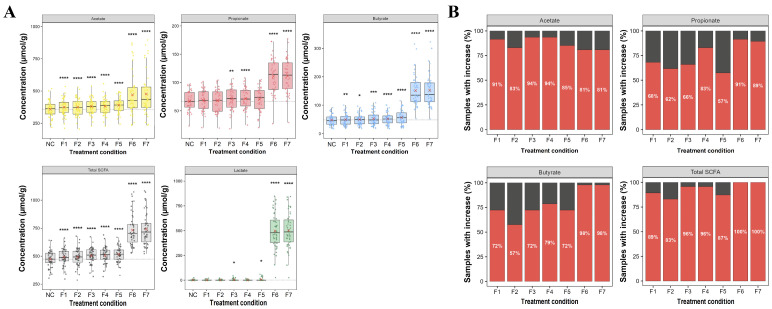
Component-level SCFAs production analysis of the Phytonutrient-enriched prebiotic mixture (PEP). (**A**) Concentration of acetate, propionate, butyrate, and lactate in Study 1. In all panels, the *x*-axis denotes the treatment condition (NC, negative control; F1–F7, individual PEP compositions) and the *y*-axis denotes metabolite concentration (µmol/g) in (**A**) or the proportion of samples showing an increase relative to NC (%) in (**B**). F1–F7 correspond to the PEP compositions detailed in Table S1: F1, heat-treated probiotic component; F2, micronutrients (vitamins and zinc); F3–F5, phytonutrient-rich components (F3, herbs and spices; F4, leafy greens; F5, fruit and vegetable blends); and F6–F7, fermentable fiber-rich components (F6, fructooligosaccharides; F7, fructooligosaccharides plus apple fiber). Total SCFAs were defined as the sum of acetate, propionate, and butyrate. (**B**) Proportion of samples exhibiting increased metabolite levels relative to the negative control (NC) (>0, red); the remaining fraction represents no increase (≤0, black). Asterisks indicate statistical significance compared with NC based on Benjamini–Hochberg (BH)-adjusted paired Wilcoxon signed-rank tests (* q < 0.05, ** q < 0.01, *** q < 0.001, **** q < 0.0001).

To characterize microbiome-derived metabolic responses to PEP components, an ex vivo evaluation was performed using individual fecal samples from healthy donors treated with seven different PEP compositions (Figure 2). The tested compositions represented distinct compositional features of the PEP framework described in Table S1, including heat-treated probiotic components (F1), micronutrients (F2), phytonutrient-rich components derived from herbs/spices, leafy greens, and fruit–vegetable blends (F3–F5), and fermentable fiber-rich components containing fructooligosaccharides and apple fiber (F6–F7). All compositions were applied based on daily intake-equivalent conditions to enable comparative assessment of their effects on microbiome-derived metabolite production.

Across donors, PEP compositions differentially modulated acetate, propionate, butyrate, total SCFAs, and lactate production relative to the negative control (NC) (Figure 2A). Overall, acetate, butyrate, and total SCFAs showed consistent and statistically significant increases across all compositions compared with NC, indicating an overall increase in microbial metabolic activity. In contrast, propionate exhibited a more selective response pattern, with significant increases primarily observed in F3 and F4. Similarly, lactate production showed a distinct pattern, with significant increases detected in F3 and F5, while other compositions showed minimal or no significant changes. In addition to these metabolite-specific differences, the magnitude of response varied across composition types. The fermentable fiber-enriched compositions (F6 and F7) induced the largest increases in acetate, butyrate, and total SCFAs, consistent with their role as key substrates for microbial metabolism. Phytonutrient-containing compositions (F3–F5) showed more moderate but metabolite-specific effects, particularly influencing propionate and lactate production. These patterns indicate that different components within the PEP framework contribute to distinct metabolic outputs.

Responder analysis further supported the consistency of these effects across individual fecal microbiomes (Figure 2B). The proportions of samples exhibiting increased acetate, propionate, butyrate, and total SCFAs production were generally high across compositions, with the highest responder rates observed for butyrate and total SCFAs in F6 and F7. In addition to individual metabolite profiles, total acids (defined as the sum of acetate, propionate, butyrate, and lactate) were significantly increased across all compositions relative to NC, with the largest increases observed in F6 and F7 (Figure S1A). Furthermore, the butyrogenic conversion index (BCI) showed a small but statistically significant increase in the fiber-rich components F6 and F7 relative to the negative control (Figure S1B), with the remaining components remaining close to the control level; this indicates a modest shift in the relative acetate/lactate balance under these fiber-rich conditions.

Collectively, these findings define the metabolic response landscape of PEP compositions in the ex vivo system and demonstrate that while overall metabolic activity is broadly enhanced, individual metabolites exhibit composition-dependent response patterns.

### 3.2. Interaction Between PEP and Probiotics Enhances Microbiome-Derived Metabolic Responses

**Figure 3:**
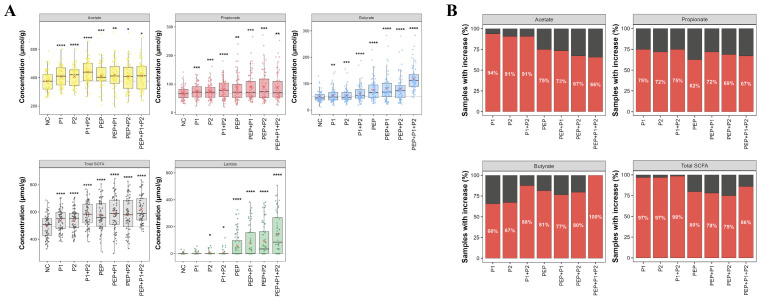
Analysis of SCFA production following combined treatment of the phytonutrient-enriched prebiotic mixture (PEP) and probiotics. (**A**) Concentrations of acetate, propionate, butyrate, and lactate measured in Study 2. Total SCFAs were defined as the sum of acetate, propionate, and butyrate. (**B**) Proportion of samples exhibiting increased metabolite levels relative to the negative control (NC) (>0, red); the remaining fraction represents no increase (≤0, black). Asterisks indicate statistical significance compared with NC based on Benjamini–Hochberg (BH)-adjusted paired Wilcoxon signed-rank tests (* q < 0.05, ** q < 0.01, *** q < 0.001, **** q < 0.0001).

To investigate whether combined treatment of PEP and probiotics enhances microbiome-derived metabolic responses, an ex vivo study was conducted using two probiotic products (P1 and P2), applied alone or in combination with PEP (Figure 3). The experimental design included single treatments (PEP, P1, P2), probiotic combinations (P1+P2), and combined treatments (PEP+P1, PEP+P2, and PEP+P1+P2), enabling direct comparison between individual and combined conditions.

A clear enhancement was observed under combined treatment conditions. Across donors, all treatments increased acetate, propionate, butyrate, total SCFAs, and lactate relative to the negative control (NC); however, the magnitude of response was consistently greater when PEP and probiotics were applied together (Figure 3A). This difference was most pronounced for butyrate and total SCFAs, which showed the highest levels in PEP-containing combinations, particularly in the PEP+P1+P2 group, whereas single probiotic treatments produced comparatively modest changes. Lactate exhibited a distinct pattern that further differentiated treatment effects. While increases were observed across conditions, lactate levels were substantially higher in PEP-containing treatments and were further elevated under combined conditions compared with probiotic-only treatments. The consistency of these interaction effects across individual microbiomes was supported by responder analysis (Figure 3B). Combined treatments showed higher proportions of samples with increased metabolite production than single treatments, with particularly strong responder rates for butyrate and total SCFAs in PEP-containing combinations. At the aggregate level, total acids increased significantly under PEP-containing treatments, particularly in combined PEP and probiotic treatments (Figure S2A). The butyrogenic conversion index did not differ significantly among conditions and remained comparable to the negative control (Figure S2B). Collectively, these results indicate that combined treatment of PEP and probiotics produces greater microbiome-derived metabolic responses than individual treatments, with pronounced effects on butyrate and total SCFAs.

### 3.3. Clinical Validation of PEP and Probiotic Effects on Microbiome-Derived Metabolites and Bowel Function

A randomized, parallel-group clinical study was conducted to examine whether the metabolic patterns observed ex vivo are reflected in human subjects. Participants were assigned to one of three primary groups—PEP alone, probiotics alone, or combined PEP and probiotic treatment—and followed for 4 weeks.

Overall, increases in microbiome-derived metabolites were observed across all treatment groups (Table 2). The combined PEP and probiotic group showed numerically larger changes in butyrate and total SCFAs, though these differences should be interpreted in the context of within-group comparisons rather than as evidence of between-group superiority. This pattern is consistent with the ex vivo findings (Section 3.2), where combined PEP and probiotic treatments showed enhanced microbiome-derived metabolic responses compared with individual treatments. This trend was most evident for butyrate and total SCFAs, which exhibited larger increases under combined treatment compared with either intervention alone. Acetate concentrations increased significantly across all groups, with mean increases of 11.2 µmol/g in the PEP group, 13.9 µmol/g in the probiotic group, and 15.0 µmol/g in the combined group. Propionate showed relatively modest changes, with increases of 2.9 µmol/g (PEP), 2.5 µmol/g (probiotics), and 1.9 µmol/g (combined). In contrast, butyrate demonstrated clearer group-dependent differences, increasing by 4.4 µmol/g in the PEP group and 5.2 µmol/g in the probiotic group, compared with a greater increase of approximately 6 µmol/g in the combined group (*p* < 0.05). A similar trend was observed for total SCFAs, which increased by 18.6 µmol/g in the PEP group and 21.7 µmol/g in the probiotic group, while reaching 22.9 µmol/g in the combined group (*p* < 0.05).

Bowel function outcomes, assessed using the Bristol Stool Scale (Table 3), showed a consistent shift toward normal stool types (Types 3–5) across all groups. Participants with constipation-type baseline stool patterns (Types 1–2) transitioned to the normal range in all groups. Improvements were also observed among participants with diarrheal baseline patterns (Types 6–7), with complete normalization in the PEP and combined groups.

Taken together, these findings indicate that while both PEP and probiotics independently improve microbiome-derived metabolite production and bowel function, their combined administration results in greater and more consistent effects, particularly for butyrate and total SCFAs, suggesting that combined administration may lead to greater microbiome-derived metabolite production than either intervention alone.

## 4. Discussion

The present study demonstrates that a phytonutrient-enriched prebiotic mixture (PEP) enhances microbiome-derived metabolic activity, and that its combination with probiotics was associated with larger metabolic responses than either intervention alone. These effects were most evident for butyrate and total SCFA production and were consistently observed across both ex vivo and clinical settings. In Study 1, metabolite profiling across multiple PEP compositions showed that acetate, butyrate, and total SCFAs were broadly increased, whereas propionate and lactate exhibited more composition-dependent responses. This pattern suggests that distinct components within PEP differentially influence microbial metabolic routing. In Study 2, combined PEP–probiotic treatments, evaluated across multiple probiotic conditions, resulted in higher metabolite levels and greater responder proportions compared with individual treatments, particularly for butyrate and total SCFAs, indicating that fermentable substrates and probiotics act cooperatively rather than independently. In Study 3, these interaction patterns were recapitulated in the clinical setting, where the combined group, representing pooled probiotic-containing conditions, showed the largest increases in butyrate and total SCFAs along with improved stool patterns. Together, these findings demonstrate consistent patterns between the ex vivo and clinical studies.

A mechanistic interpretation of these results is supported by the complementary roles of PEP components. Fermentable fibers such as fructooligosaccharides and apple fiber likely serve as primary substrates for saccharolytic fermentation and SCFA production, particularly for acetate and butyrate [19]. However, the enhanced responses observed in fiber-enriched PEP compositions and in PEP-containing combined treatments suggest that substrate availability alone does not fully account for the observed metabolic patterns [20]. Phytonutrients may modulate microbial community structure and metabolic efficiency by influencing substrate accessibility, redox balance, and interspecies interactions. In particular, polyphenol-rich compounds have been associated with shifts in SCFA production in both experimental and clinical contexts [21]. These observations support a model in which the fermentable fraction of PEP provides the metabolic foundation, while the phytonutrient fraction modulates community-level fermentation dynamics, increasing the likelihood of downstream butyrate production [13]. These observations are consistent with a growing body of work on phytonutrients and synbiotic combinations. Plant polyphenols and phytochemicals such as those in ginger, turmeric, and mixed fruit and vegetable extracts have repeatedly been shown to be biotransformed by the gut microbiota and to shift microbial fermentation toward increased SCFA output [2,21], and our group has previously reported that a multivitamin and mineral supplement containing phytonutrients exerted measurable antioxidant effects in a randomized controlled trial [4]. Likewise, substrate-guided synbiotic approaches that deliberately match fermentable substrates to microbial metabolic capacity have been proposed as a more rational route to enhanced metabolite production than single-component interventions [7]. The present findings extend these reports by showing, within a single personalized ex vivo-to-clinical framework, that pairing a phytonutrient-enriched prebiotic matrix with probiotics produces greater butyrate- and SCFA-directed responses than either component alone.

Further support for this network-level interpretation is provided by the preferential increase in butyrate and the behavior of lactate and the butyrogenic conversion index. Butyrate is often produced through secondary cross-feeding pathways rather than as a primary fermentation end product. In this process, intermediate metabolites such as lactate and acetate are utilized by butyrate-producing bacteria [13,22]. Several human gut microbes, including *Eubacterium hallii* and *Anaerobutyricum*/*Anaerostipes* species, are known to convert lactate and acetate into butyrate [23]. Accordingly, lactate should be viewed not only as an end product but also as a key metabolic intermediate that supports downstream butyrate formation under stable microbial conditions [24]. It should be noted, however, that the accumulation of lactate observed in this study may partly reflect the closed nature of the PMAS ex vivo system. Unlike the in vivo gut environment, where continuous absorption, flow, and microbial turnover facilitate the further utilization of intermediate metabolites, the closed ex vivo setting may allow transient intermediates such as lactate to accumulate and be detected before complete metabolic conversion. Within this context, the butyrogenic conversion index used in the present study can be interpreted as a systems-level indicator of the community’s capacity to channel acetate- and lactate-associated upstream fermentation activity toward downstream butyrate production, rather than as a universal biochemical constant [23,25]. The marked increase in this index in the fiber-enriched compositions and in PEP-containing combination treatments is therefore consistent with a shift in metabolic balance toward butyrate-forming cross-feeding. This interpretation also helps explain why lactate elevations in PEP-containing conditions should not necessarily be viewed negatively, but instead may reflect an expanded precursor pool supporting downstream butyrate output.

A major strength of this study is the use of the Personalized Pharmaceutical Meta-Analytical Screening (PMAS) platform as a translational bridge between ex vivo screening and clinical validation. PMAS preserves inter-individual microbiome variability while enabling high-throughput evaluation of multiple treatments under controlled anaerobic conditions [16]. This is particularly valuable in microbiome-targeted nutrition, where individual variability can strongly influence treatment response and where direct clinical testing of all candidate formulations is impractical [26]. Notably, previous work using the same platform successfully identified *Lactobacillus fermentum* HEM20792 as a therapeutic candidate in COPD models, demonstrating that PMAS-derived findings can translate into functional outcomes beyond in vitro metabolic measurements [16]. The present study extends this concept to nutritional interventions, showing that PMAS can guide the identification of promising PEP–probiotic combinations by enabling systematic evaluation across diverse probiotic conditions, while supporting interpretation of overall probiotic effects at the translational level with higher clinical relevance.

The clinical findings further support this translational framework. Although the clinical study was limited to three treatment groups, the combined PEP and probiotic group showed the most consistent increases in butyrate and total SCFAs, along with improved stool patterns. These findings are consistent with the known physiological roles of butyrate in colonocyte energy metabolism, epithelial barrier integrity, and immune regulation [27]. Collectively, these changes indicate that the intervention increased fecal metabolites associated with beneficial gut microbial activity, in particular butyrate and total SCFAs. The known physiological roles of butyrate in colonocyte energy metabolism and epithelial integrity provide a plausible biological rationale for why such increases may be favorable for supporting intestinal barrier homeostasis and broader gut health. Confirmation of whether these metabolite-level changes translate to measurable host benefits will require future studies incorporating dedicated functional and mechanistic endpoints. At the same time, the relatively modest and variable response of propionate suggests that not all microbial metabolites respond uniformly to combined treatment, likely reflecting differences in metabolic pathway complexity and microbial community dependence.

Several limitations should be considered. First, although PMAS reproduces key features of the intestinal environment, it remains a closed ex vivo system and therefore cannot fully capture host-related processes such as metabolite absorption, mucosal signaling, immune interactions, and systemic metabolism. This limitation is particularly relevant for intermediate metabolites such as lactate, whose accumulation in the ex vivo setting may partly reflect incomplete downstream utilization compared with the dynamic conditions of the in vivo gut. Second, the butyrogenic conversion index used in this study is an inference-based metric derived from metabolite relationships within the ex vivo system. While it is mechanistically grounded in known acetate–lactate-to-butyrate pathways, it should be interpreted as a systems-level indicator of metabolic potential rather than a direct measure of pathway flux. Third, the clinical study was designed to evaluate the overall effects of PEP, probiotics, and their combination in a pragmatic setting. Although multiple probiotic types were included to reflect product variability, analyses were performed by pooling probiotic-containing groups to focus on the general effects of probiotics rather than product-specific differences. As a result, the study was not intended to resolve formulation-specific or strain-level effects, which may require more targeted study designs. Another important limitation is the absence of a placebo or non-intervention control group in the clinical study. Consequently, although increases in fecal metabolites and improvements in stool patterns were observed over the intervention period, the study design does not allow definitive attribution of these changes solely to the administered products. Potential influences of temporal variation, dietary changes, behavioral factors, and regression to the mean cannot be fully excluded. Future placebo-controlled studies will be necessary to establish causality and quantify the magnitude of intervention-specific effects. Finally, this study focused primarily on fermentation-derived metabolites and bowel function as functional readouts. Future studies incorporating metagenomic profiling, pathway-resolved analyses, or isotope-based metabolic tracing would provide deeper insight into the microbial taxa and biochemical pathways driving the observed enhanced, butyrate-centered responses under combined treatment.

Overall, this study supports a model in which PEP provides a phytonutrient-enriched fermentable substrate environment, probiotics contribute complementary functional capacity, and their combination promotes a shift in microbial metabolism toward butyrate-centered outputs. These findings integrate ex vivo screening, synbiotic interaction analysis, and clinical validation, highlighting a translational framework for microbiome-targeted nutritional strategies.

## 5. Conclusions

This study demonstrates that a phytonutrient-enriched prebiotic mixture (PEP) enhances microbiome-derived metabolic activity and that its combination with probiotics leads to greater and more consistent increases in butyrate and total SCFAs across both ex vivo and clinical settings. These findings suggest that combined administration of PEP and probiotics may enhance microbiome-derived metabolic activity compared with either intervention alone. The observed patterns further suggest that this effect is associated with enhanced cross-feeding interactions, reflected by increased butyrate production and shifts in metabolite balance toward butyrate-centered outputs, indicating a potential contribution to microbial metabolic activity and to fecal metabolite profiles associated with beneficial gut microbial activity. Importantly, the integration of the PMAS platform enabled systematic, individualized screening of microbiome responses and demonstrated translational relevance, as ex vivo findings were recapitulated in human outcomes. Together, this study provides both ex vivo and clinical support for combined microbiome-targeted nutritional interventions and presents a scalable framework for developing microbiome-targeted nutritional interventions with improved translational potential. From an applied perspective, this approach offers a practical route to next-generation prebiotic and synbiotic products, as the PMAS platform can rationally pair fermentable substrates, phytonutrients, and probiotic strains to optimize butyrate- and SCFA-directed responses and, in principle, personalize formulations to an individual’s microbiome. The same substrate-to-metabolite screening logic could, with species-specific validation, extend beyond human nutrition to companion-animal and livestock feed applications.

## Figures and Tables

**Table 1:** Baseline characteristics of the fecal donors.

	Study 1		Study 2		Study 3		*p*
Characteristic	Estimate	SD	Estimate	SD	Estimate	SD	
Sex (M/F, M%)	18/29 (38.3%)	-	23/41 (35.9%)	-	37/44 (45.7%)	-	0.486 ^†^
Age (years)	37.5	20.5	37.8	21.3	44.3	12.8	0.413 ^‡^
BMI (kg/m^2^)	23.8	8.3	22.5	5.2	22.9	3.1	0.624 ^‡^

The estimates of age and BMI represent the mean values. SD: Standard Deviation. ^†^ Fisher’s exact test. ^‡^ Kruskal–Wallis rank-sum test, two-sided.

**Table 2:** Changes in fecal SCFA levels following PEP, probiotic, and combined treatments in clinical study.

	PEP (*n* = 15)						Probiotics (*n* = 33)						PEP+Probiotics (*n* = 33)					
	Pre		Post		Δ	*p* ^†^	Pre		Post		Δ	*p* ^†^	Pre		Post		Δ	*p* ^†^
SCFA	Mean	SD	Mean	SD			Mean	SD	Mean	SD			Mean	SD	Mean	SD		
Acetate	50.6	22.1	61.8	27.9	11.2	**0.047**	52.2	22.3	66.1	28.9	13.9	**0.041**	55	17.4	69.9	26.9	15.0	**0.011**
Propionate	17.3	9.1	20.2	7.1	2.9	0.085	18.4	9.6	21	7.7	2.5	0.253	18.2	9.8	20.2	9.3	1.9	0.408
Butyrate	10.8	4.7	15.2	10.3	4.4	**0.032**	11.1	7.0	16.3	11.8	5.2	**0.031**	11.5	7.8	17.5	13.4	6.0	**0.030**
Total SCFA	78.7	33.8	97.2	39.3	18.6	**0.013**	81.7	36.3	103.4	42.1	21.7	**0.033**	84.7	29.4	107.6	45.4	22.9	**0.024**

Data are presented as mean ± standard deviation (SD) values. Δ represents the mean difference between post- and pre-intervention values (Post-Pre). ^†^ Paired *t*-test (Pre vs. Post). Bold values represent statistically significant differences (*p* < 0.05).

**Table 3:** Changes in Bristol Stool Scale categories from pre- to post-intervention across treatment groups in Study 3.

Pre Bristol Category	Post Bristol Category	PEP	Probiotics	PEP+Probiotics
Constipated (Type 1–2)	Constipated	0 (0.0%)	0 (0.0%)	0 (0.0%)
	Normal	3 (100.0%)	6 (100.0%)	6 (100.0%)
	Diarrheal	0 (0.0%)	0 (0.0%)	0 (0.0%)
Normal (Type 3–5)	Constipated	0 (0.0%)	3 (12.5%)	1 (3.8%)
	Normal	10 (90.9%)	21 (87.5%)	23 (88.5%)
	Diarrheal	1 (9.1%)	0 (0.0%)	2 (7.7%)
Diarrheal (Type 6–7)	Constipated	0 (0.0%)	0 (0.0%)	0 (0.0%)
	Normal	1 (100.0%)	2 (66.7%)	1 (100.0%)
	Diarrheal	0 (0.0%)	1 (33.3%)	0 (0.0%)

Data are presented as N (%). Percentages were calculated within each pre-intervention Bristol stool scale category. Bristol categories were defined as constipated (types 1–2), normal (types 3–5), and diarrheal (types 6–7).

## Data Availability

The datasets generated and/or analyzed during the current study are not publicly available due to data privacy considerations but are available from the corresponding author upon reasonable request.

## Supplementary Materials

The following supporting information can be downloaded at: https://www.mdpi.com/article/10.3390/biology15131006/s1, Figure S1: Analysis of total acid production and butyrogenic conversion in PEP components. (A) Total acids and (B) butyrogenic conversion index (BCI) measured in Study 1. Total acids were defined as the sum of total SCFAs and lactate. The BCI summarizes the relative contribution of acetate and lactate to the total organic acid pool and is used as an exploratory, inference-based index rather than a direct measure of butyrate formation or metabolic flux. The red “×” indicates the mean. Asterisks indicate statistical significance compared with the negative control (NC) based on Benjamini–Hochberg (BH)-adjusted paired Wilcoxon signed-rank tests (* q < 0.05, ** q < 0.01, *** q < 0.001, **** q < 0.0001); Figure S2: Analysis of total acid production and butyrogenic conversion following combined treatment of PEP and probiotics. (A) Total acids and (B) butyrogenic conversion index (BCI) measured in Study 2. Total acids were defined as the sum of total SCFAs and lactate. The BCI summarizes the relative contribution of acetate and lactate to the total organic acid pool and is used as an exploratory, inference-based index rather than a direct measure of butyrate formation or metabolic flux. The red “×” indicates the mean. Asterisks indicate statistical significance compared with the negative control (NC) based on Benjamini–Hochberg (BH)-adjusted paired Wilcoxon signed-rank tests (* q < 0.05, ** q < 0.01, *** q < 0.001, **** q < 0.0001); Table S1: Component of Phytonutrient-enriched prebiotic mixture (PEP) used for ex vivo profiling (Study 1); Table S2: Products and daily intake used in the ex vivo PEP–probiotic combination study (Study 2) and clinical study (Study 3).

## Abbreviations

The following abbreviations are used in this manuscript:

PEP	Phytonutrient-Enriched Prebiotic Mixture
SCFA	Short-Chain Fatty Acid
PMAS	Personalized Pharmaceutical Meta-Analytical Screening
FOS	Fructooligosaccharide
LC-RID	Liquid Chromatography-Refractive Index Detector
BCI	Butyrogenic Conversion Index

## References

[1] Plaza-Diaz J., Herrera-Quintana L., Olivares-Arancibia J., Vázquez-Lorente H. (2026). Personalized nutrition through the gut microbiome in metabolic syndrome and related comorbidities. Nutrients.

[2] Santhiravel S., Bekhit A.E.D.A., Mendis E., Jacobs J.L., Dunshea F.R., Rajapakse N., Ponnampalam E.N. (2022). The impact of plant phytochemicals on the gut microbiota of humans for a balanced life. Int. J. Mol. Sci..

[3] Lippolis T., Cofano M., Caponio G.R., De Nunzio V., Notarnicola M. (2023). Bioaccessibility and bioavailability of diet polyphenols and their modulation of gut microbiota. Int. J. Mol. Sci..

[4] Kang S., Lim Y., Kim Y.J., Jung E.S., Suh D.H., Lee C.H., Park E., Hong J., Velliquette R.A., Kwon O. (2019). Multivitamin and mineral supplementation containing phytonutrients scavenges reactive oxygen species in healthy subjects: A randomized, double-blinded, placebo-controlled trial. Nutrients.

[5] Bedu-Ferrari C., Biscarrat P., Langella P., Cherbuy C. (2022). Prebiotics and the human gut microbiota: From breakdown mechanisms to the impact on metabolic health. Nutrients.

[6] Sánchez B., Delgado S., Blanco-Míguez A., Lourenço A., Gueimonde M., Margolles A. (2017). Probiotics, gut microbiota, and their influence on host health and disease. Mol. Nutr. Food Res..

[7] Speckmann B., Ehring E., Hu J., Rodriguez Mateos A. (2024). Exploring substrate–microbe interactions: A metabiotic approach toward developing targeted synbiotic compositions. Gut Microbes.

[8] Kezer G., Paramithiotis S., Khwaldia K., Harahap I.A., Čagalj M., Šimat V., Smaoui S., Elfalleh W., Ozogul F., Esatbeyoglu T. (2025). A comprehensive overview of the effects of probiotics, prebiotics and synbiotics on the gut–brain axis. Front. Microbiol..

[9] Koh A., De Vadder F., Kovatcheva-Datchary P., Bäckhed F. (2016). From dietary fiber to host physiology: Short-chain fatty acids as key bacterial metabolites. Cell.

[10] Den Besten G., Van Eunen K., Groen A.K., Venema K., Reijngoud D.J., Bakker B.M. (2013). The role of short-chain fatty acids in the interplay between diet, gut microbiota, and host energy metabolism. J. Lipid Res..

[11] Hosseini E., Grootaert C., Verstraete W., Van de Wiele T. (2011). Propionate as a health-promoting microbial metabolite in the human gut. Nutr. Rev..

[12] Salvi P.S., Cowles R.A. (2021). Butyrate and the intestinal epithelium: Modulation of proliferation and inflammation in homeostasis and disease. Cells.

[13] Louis P., Duncan S.H., Sheridan P.O., Walker A.W., Flint H.J. (2022). Microbial lactate utilisation and the stability of the gut microbiome. Gut Microbiome.

[14] Lampe J.W., Navarro S.L., Hullar M.A., Shojaie A. (2013). Inter-individual differences in response to dietary intervention: Integrating omics platforms towards personalised dietary recommendations. Proc. Nutr. Soc..

[15] Adesina P.A., Ooka M., TeKrony C., Xia M. (2025). Emerging advances in intestinal models for in vitro preclinical research. Am. J. Physiol. Gastrointest. Liver Physiol..

[16] Kim N.H., Oh J., Lee J.H., Lee S., Jung E.S., Suh D.H., Kang H.-J., Kim B., Kim H.-S., Jung H.R. (2026). A colon mimetic screening approach reveals *Lactobacillus fermentum* as a microbiome-based therapy for COPD. npj Biofilms Microbiomes.

[17] Park S., Ji Y. (2022). Method for Screening Personalized Intestinal Environment-Improving Material and Composition Therefor. U.S. Patent.

[18] Park S., Ji Y. (2020). PMAS Method for Screening Personalized Intestinal Environment-Improving Material. KR Patent.

[19] Flint H.J., Louis P., Duncan S.H. (2024). Why does increased microbial fermentation in the human colon shift toward butyrate?. AIMS Microbiol..

[20] Moens F., Verce M., De Vuyst L. (2017). Lactate- and acetate-based cross-feeding interactions between selected strains of lactobacilli, bifidobacteria and colon bacteria in the presence of inulin-type fructans. Int. J. Food Microbiol..

[21] Mukhopadhya I., Louis P. (2025). Gut microbiota-derived short-chain fatty acids and their role in human health and disease. Nat. Rev. Microbiol..

[22] Duncan S.H., Louis P., Flint H.J. (2004). Lactate-utilizing bacteria isolated from human feces that produce butyrate as a major fermentation product. Appl. Environ. Microbiol..

[23] Detman A., Mielecki D., Chojnacka A., Salamon A., Błaszczyk M.K., Sikora A. (2019). Cell factories converting lactate and acetate to butyrate: *Clostridium butyricum* and microbial communities from dark fermentation bioreactors. Microb. Cell Fact..

[24] Honda S., Eguchi H., Okino Y., Wang D.S. (2025). The probiotic strain *Clostridium butyricum* TO-A produces butyrate by utilizing lactate and acetate. Int. J. Mol. Sci..

[25] Munoz-Tamayo R., Laroche B., Walter E., Dore J., Duncan S.H., Flint H.J., Leclerc M. (2011). Kinetic modelling of lactate utilization and butyrate production by key human colonic bacterial species. FEMS Microbiol. Ecol..

[26] Hughes R.L., Kable M.E., Marco M., Keim N.L. (2019). The role of the gut microbiome in predicting response to diet and the development of precision nutrition models. Part II: Results. Adv. Nutr..

[27] Parada Venegas D., De la Fuente M.K., Landskron G., González M.J., Quera R., Dijkstra G., Harmsen H.J.M., Faber K.N., Hermoso M.A. (2019). Short chain fatty acids (SCFAs)-mediated gut epithelial and immune regulation and its relevance for inflammatory bowel diseases. Front. Immunol..

