# Supplementary material for: Phytonutrient-Enriched Prebiotic Mixture Primes the Gut Environment to Enhance Probiotic Efficacy: Ex Vivo Screening and a Human Clinical Trial
Source: Biology (Basel). 2026 Jun 25;15(13):1006. doi: 10.3390/biology15131006 (PMC13360448; doi:10.3390/biology15131006)
Supplement: Supplementary file 1 [file biology-15-01006-s001.zip › Figure S1.pdf]

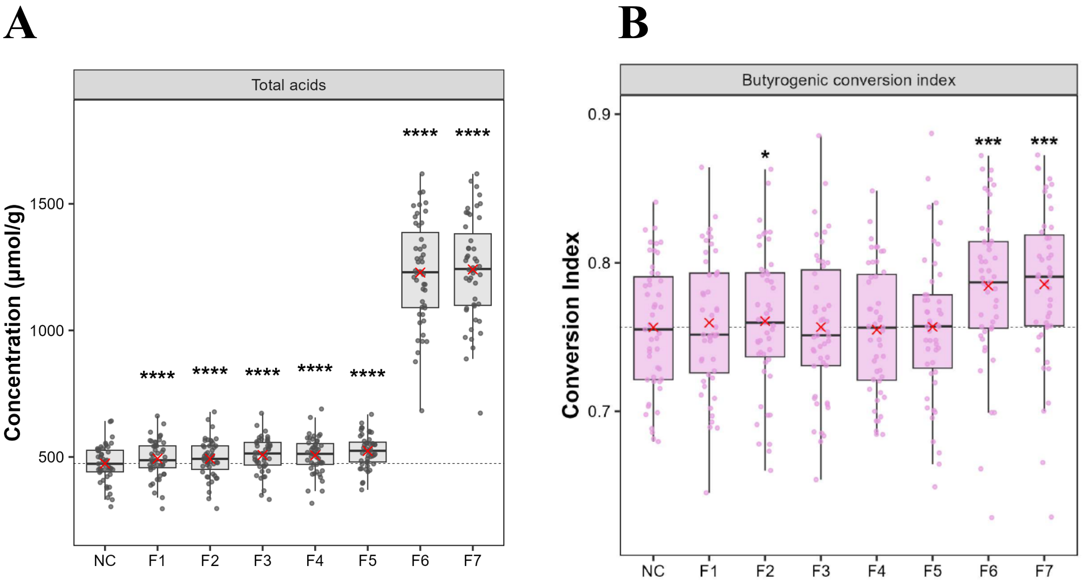

Figure S1: Analysis of total acid production and butyrogenic conversion in PEP components. (A) Total acids and (B) butyrogenic conversion index (BCI) measured in Study 1. Total acids were defined as the sum of total SCFAs and lactate. The BCI summarizes the relative contribution of acetate and lactate to the total organic acid pool and is used as an exploratory, inference-based index rather than a direct measure of butyrate formation or metabolic flux. The red “×” indicates the mean. Asterisks indicate statistical significance compared with the negative control (NC) based on Benjamini–Hochberg (BH)-adjusted paired Wilcoxon signed-rank tests (* $q < 0.05$, ** $q < 0.01$, *** $q < 0.001$, **** $q < 0.0001$)
